# Reparación de tejidos perirradiculares en el tratamiento endodóntico no quirúrgico. Una revisión

**DOI:** 10.21142/2523-2754-1203-2024-210

**Published:** 2024-09-17

**Authors:** José Alberto Castillo Páez, Marietta Álvarez

**Affiliations:** 1 Departamento de Estomatoquirúrgica, Facultad de Odontología, Universidad de Carabobo. Valencia, Venezuela. josecastillo031285@gmail.com , alvarezmarietta@gmail.com Universidad de Carabobo Departamento de Estomatoquirúrgica Facultad de Odontología Universidad de Carabobo Valencia Venezuela josecastillo031285@gmail.com alvarezmarietta@gmail.com

**Keywords:** lesión periapical, tejidos periapicales, reparación biológica, reparación apical, periapical lesion, periapical tissues, biological repair, apical repair

## Abstract

**Introducción:**

El tratamiento endodóntico no quirúrgico tiene como finalidad la prevención de la periodontitis apical, lo que se consigue con la desinfección del sistema de conductos radiculares. Adicionalmente, existen patologías donde el contenido tóxico del conducto va a los tejidos perirradiculares y ocasiona osteólisis o lesiones del tejido óseo que radiográficamente se observan como imágenes radiolúcidas. Cuando el tratamiento endodóntico es exitoso, la reparación de estas lesiones ocurre con el transcurrir del tiempo.

**Objetivo:**

Analizar el proceso de reparación perirradicular del tratamiento endodóntico no quirúrgico y los factores que inciden en este.

**Materiales y métodos:**

Se realizó una búsqueda electrónica por los buscadores PubMed, Scopus, Google Académico y SciELO, con las palabras “Non-Surgical Endodontic Treatment”, “Periapical Tissues”, “Biological Repair” y “Apical Repair”. Se consideraron factores como textos incompletos, textos en PDF y fecha de publicación del artículo, que comprendió datas de los últimos cinco (05) años.

**Resultados:**

La información revisada estuvo comprendida por 236 artículos que fueron analizados con los criterios de inclusión y exclusión, y solo cumplieron estos criterios 42 de ellos.

**Conclusión:**

El éxito del tratamiento endodóntico radica en la ausencia de signos y síntomas clínicos y radiográficos de infección, lo que se consigue con una buena aplicación de protocolos y procedimientos clínicos enfocados en la desinfección del sistema de conductos radiculares, desde el diagnóstico, la preparación biomecánica y desinfección del sistema de conductos radiculares con las sustancias irrigantes y la obturación, incluida la rehabilitación coronal desde el punto de visto estético o protésico.

## Abstract (en)

**Introduction:**

Non-surgical endodontic treatment aims to prevent apical periodontitis, achieved with the disinfection of the root canal system. Additionally, there are pathologies where the toxic content of the canal goes to the periradicular tissues, causing osteolysis or lesions of the bone tissue that are radiographically observed as radiolucent images. When the endodontic treatment is successful, the repair of these lesions occurs over time.

**Aim:**

To analyze the periradicular repair process of non-surgical endodontic treatment and the factors that affect it.

**Materials and Methods:**

An electronic search was carried out using the search engines PUBMED, Scopus, Google Academic, and Scielo with the words “Non-Surgical Endodontic Treatment,” “Periapical Tissues,” “Biological Repair,” “Apical Repair.” Factors such as incomplete texts, PDF texts, and publication date of the article were considered, including data from the last five (05) years.

**Results:**

The information reviewed comprised 236 articles analyzed with the inclusion and exclusion criteria, and only 42 articles met these criteria.

**Conclusion:**

The success of endodontic treatment lies in the absence of clinical and radiographic signs and symptoms of infection; this is achieved with a good application of clinical protocols and procedures focused on the disinfection of the root canal system, from diagnosis, biomechanical preparation, and disinfection of the root canal system with irrigating substances and filling, including coronal rehabilitation from an aesthetic and/or prosthetic point of view.

## INTRODUCCIÓN

El tratamiento endodóntico no quirúrgico es ese procedimiento clínico que tiene como fin u objetivo principal el tratamiento y prevención de la periodontitis apical; asimismo, es realizado para tratar patologías pulpares irreversibles y patologías de los tejidos periapicales ^1^. Cuando se realiza este procedimiento clínico, se busca realizar una desinfección del sistema de conductos radiculares lo más completa o efectiva posible, tornando inviable el ambiente intraconducto para los microorganismos y así evitar el crecimiento y proliferación bacteriana, mediante el uso de sustancias irrigadoras que tienen propiedades antibacterianas, y en consecuencia favorecer la reparación de los tejidos periapicales ^2^.

De esta manera, puede agregarse que el tratamiento endodóntico no quirúrgico está indicado en casos en los que la pulpa se encuentra en un estado inflamatorio irreversible, que clínicamente, según la Sociedad Americana de Endodoncia, se identifican como pulpitis irreversible sintomática y pulpitis irreversible asintomática. Además, está indicado en casos de muerte o necrosis del tejido pulpar ^3^. 

Adicionalmente, existen patologías de los tejidos periapicales para los cuales el tratamiento endodóntico no quirúrgico está indicado, estas son la periodontits apical sintomática y asintomática, los abscesos periapicales agudos y crónicos, y la osteítis condensante ^3^.

Ahora bien, una de las características principales de algunas de las patologías perirradiculares en las que está indicado el tratamiento endodóntico es la presencia de imágenes radiolúcidas periapicales, las cuales se producen como consecuencia de la osteólisis derivada de la acción de bacterias y sus toxinas que pasan del conducto radicular a los tejidos periapicales, obviamente tras una necrosis pulpar. En ellas se evidencia la respuesta inmune del organismo ante la acción bacteriana, ya que es gracias a ella se “encapsulan” los procesos infecciosos, lo que produce lesiones a nivel del periápice y que posteriormente se evidenciarán radiográficamente como imágenes radiolúcidas ^4^^,^^5^.

Cuando se realiza un tratamiento endodóntico no quirúrgico en presencia de estas lesiones perirradiculares, es esencial e imperativa una buena desinfección del sistema de conductos radiculares, lo que se logra con una buena preparación bioquímicomecánica que incluye no solo la preparación con instrumentos manuales o rotatorios, sino también con el buen uso de los agentes irrigantes, que juegan un rol fundamental en la desinfección ^5^. 

Igualmente, si se logra una buena desinfección, limpieza y desbridamiento del sistema de conductos radiculares, se estarían erradicando una gran cantidad de toxinas y bacterias, o se estaría creando un ambiente totalmente inviable para su crecimiento dentro del conducto, lo que favorecería la reparación de los tejidos periapicales y la disipación de las lesiones en esta zona del diente, y así se repararían los tejidos perirradiculares a largo plazo ^6^. Así, en la presente revisión se tiene como objetivo analizar el proceso de reparación perirradicular del tratamiento endodóntico no quirúrgico y los factores que inciden en él.

## MATERIALES Y MÉTODOS

Esta investigación es el resultado de información obtenida mediante la búsqueda electrónica a través de los buscadores PubMed, Scopus, Google Académico y SciELO, con las palabras “Non-Surgical Endodontic Treatment”, “Periapical Tissues”, “Biological Repair” y “Apical Repair”. En la búsqueda, se consideraron factores como textos incompletos, textos en PDF, y fecha de publicación del artículo, que comprendió datas de los últimos cinco (5) años. Los criterios de exclusión para las investigaciones encontradas fueron cartas al director, noticias, comentarios, resúmenes, investigaciones no primarias y editoriales. Los artículos fueron revisados a partir de sus títulos y resúmenes, y posteriormente se leyeron los textos completos. Como criterio de inclusión se tuvo que estos guardaran relación directa con el objetivo de la investigación, es decir, analizar el proceso de reparación perirradicular del tratamiento endodóntico no quirúrgico y los factores que inciden en él; además, se seleccionaron trabajos de tipo revisiones sistemáticas, metaanálisis, trabajos de campo, investigaciones in vitro, reportes de caso y estudios de cohorte, y se obtuvo finalmente los 42 artículos que componen las referencias de la presente revisión.

## RESULTADOS

**Figura 1:**
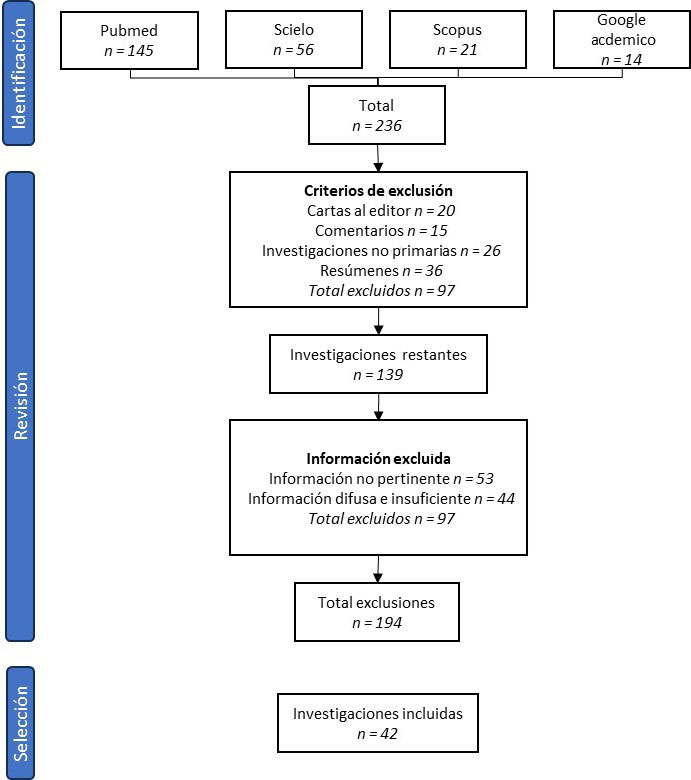
Diagrama de flujo de la recolección y selección de la información

Se revisaron 236 artículos mediante búsqueda en los diferentes buscadores *online* que conformaron la base de datos inicial. En el diagrama de flujo presentado en la Figura 1, se detallan 145 estudios de PubMed, 56 estudios de SciELO, 21 de Scopus y 14 de Google Académico. Del total de 236 investigaciones, se excluyeron 20 cartas al editor, 15 comentarios, 26 investigaciones no primarias y 36 resúmenes, para un total de 97 investigaciones. Los 139 artículos restantes fueron nuevamente revisados y se excluyeron otros 97, 53 por que no contenían información pertinente para el objetivo de esta investigación y 44 por que la información era difusa e insuficiente. Así quedaron los 42 artículos incluidos en esta revisión.

### La lesión periapical

La inflamación de los tejidos perirradiculares, para estudios como el de Luo *et al*. ^7^, inicia como una respuesta inmune o fisiológica del cuerpo ante la presencia de infección o contenido tóxico dentro del sistema de conductos radiculares; esta tiene la finalidad de evitar el pase de esas bacterias o agentes nocivos al resto del organismo, es decir, “contiene” el factor etiológico y evita su diseminación sistémica.

Así, puede mencionarse que ese pase de bacterias y contenido tóxico del conducto a los tejidos periapicales dependerá del grado de infección, que Gao *et al*. ^8^ clasifican de la siguiente forma: grado I, conducto limpio; grado II, conducto no infeccioso; grado III, conducto infeccioso, y grado IV, conducto severamente infeccioso. Entonces, grados altos de infección y contenido tóxico dentro del conducto, como bien mencionan Xu *et al*. ^9^, producen una respuesta inflamatoria que continúa o tienen como consecuencia una lesión osteolítica que, radiográficamente, se observa como una imagen radiolúcida en el periápice dental.

Al respecto, Ordinola *et al*. ^10^ señalan que el daño periapical puede clasificarse en 4 estadios:

• Estadio 1: afección sin complicaciones ni problemas, de mínima gravedad, incluyendo la ausencia o cambios mínimos en los tejidos periapicales.
• Estadio 2: complicaciones limitadas al área periapical, riesgo significativamente mayor de complicaciones con respecto a la etapa 1. El aumento en el tamaño de la lesión > 5 mm y la presencia de factores agravantes, como la complejidad del diente y la presencia de tracto sinuoso podrían estar en esta categoría.
• Estadio 3: pérdida ósea extensa más allá de la región apical inmediata. Defectos óseos completos, radiolucideces grandes, defectos de furca e imágenes en forma de J.
• Estadio 4: pérdida de dientes o desarrollo de complicaciones graves. El pronóstico en estos casos es reservado _^(10)^_ .

Todo lo descrito, como agregan Ricucci *et al*. ^11^, da a entender que, dentro del sistema de conductos radiculares, especialmente en la porción apical, existe un porcentaje alto de presencia bacteriana y de toxinas, que si no se eliminan por la vía ortógrada, es decir, por medio del tratamiento endodóntico no quirúrgico, la infección puede pasar a vía sistémica o empeorar el pronóstico de permanencia de ese diente en boca.

Y bien dicen Chandra *et al*. ^12^ que, cuando el tratamiento endodóntico no quirúrgico es exitoso, desde una visión teórica, se debe a que hubo eliminación de microbios o cuerpos extraños y, desde una visión clínica, existe ausencia de infección periapical, obturación radicular bien condensada, obturación radicular extendida hasta 1 o 2 mm dentro del ápice radiográfico, restauración coronal satisfactoria y uso de un dique de goma durante el tratamiento. 

Rao *et al*. ^13^ mencionan, además, que los éxitos o fracasos endodónticos, considerando los factores señalados, pueden establecerse tras 1 a 3 años posteriores al tratamiento, que es cuando se aprecia, tanto clínica como radiográficamente, ausencia de sintomatología, es decir, silencio clínico y reparación de las rarefacciones apicales. Añaden que la obturación es un factor clave y esencial en el éxito de un tratamiento, y que el sellado coronal o una buena rehabilitación coronal también son esenciales en el éxito y pronóstico del tratamiento endodóntico.

### La preparación biomecánica

Eliasz *et al*. ^14^ señalan que la finalidad de la preparación biomecánica en el tratamiento endodóntico no quirúrgico va más allá de remover de forma irreversible los agentes dañinos para los tejidos orgánicos pulpares y perirradiculares, sino que tiene como objetivo dar forma al sistema de conductos radiculares, manteniendo su anatomía, para facilitar la llegada de las sustancias irrigantes y de los materiales y cementos de obturación a todo el conducto, especialmente la porción apical. 

En adición, Jiang *et al*. ^15^ mencionan que dicha preparación es realizada con instrumentos de acero inoxidable estandarizados, los cuales se introducen de forma secuencial en el conducto radicular, y se hace uso de diferentes técnicas para lograr estos objetivos. En la actualidad, a estos instrumentos, además, se les añaden otros de níquel titanio que poseen características y propiedades que facilitan las maniobras dentro del conducto, lo que favorece esa conformación necesaria para lograr una buena desinfección. 

En este aspecto, se considera que la conformación, según estudios de Martins *et al*. ^16^ y Del Fabbro *et al*. ^17^, bien sea con instrumentos de acero inoxidable o de níquel-titanio, se adapta al hecho de que existen variaciones anatómicas del sistema de conductos radiculares que no permiten que la conformación sea de una única forma para todos los conductos. Además, es importante reconocer que la conformación por sí misma no logra una desinfección del sistema de conductos radiculares, sino que crea en el conducto las condiciones ideales para la llegada de las sustancias irrigantes y los materiales de obturación.

### La irrigación del sistema de conductos radiculares

La irrigación, como dicen Haapsalo *et al*. ^18^, es uno de los elementos más importantes del tratamiento endodóntico no quirúrgico, porque con ella se facilita la destrucción y remoción de microorganismos, tejido necrótico e inflamado, y eliminación del barrillo dentinario; de hecho, se busca que un irrigante sea de bajo costo, que tenga acción de barrido, reduzca la fricción de los instrumentos y mejore su capacidad de corte, controle la temperatura, disuelva materia orgánica e inorgánica, tenga acción bactericida, no sea citotóxico y no debilite la dentina ^18^. Tonini *et al*. ^19^ añaden que son todas estas propiedades las que garantizan la reparación de tejidos perirradiculares y previenen la periodontitis apical después del tratamiento endodóntico.

Por otro lado, Rath *et al*. ^20^, Cecchin *et al*. ^21^ y Prado *et al*. ^22^ señalan que las sustancias irrigantes pueden ser variadas, desde el tradicional hipoclorito de sodio, hasta el ácido etilendiaminotetracético (EDTA), el ácido cítrico, el ácido maleico o la solución fisiológica, pero su uso puede variar según el protocolo de irrigación utilizado para la patología pulpar o periapical, y la morfología del conducto radicular a tratar; adicionalmente, se consideran las propiedades del irrigante y su efecto sobre la dentina. Así puede mencionarse que el EDTA y el ácido cítrico, por ejemplo, son excelentes sustancias quelantes, de uso ideal en conductos atrésicos y de difícil acceso a porciones apicales, pero con pocas propiedades antimicrobianas.

De esta manera, Siqueira *et al*. ^23^ concuerdan en que aún hoy se tiene la concepción de que el hipoclorito de sodio es el irrigante ideal, ya que este, a pesar de ser citotóxico y tener un olor desagradable, tiene una excelente actividad antimicrobiana, disuelve materia orgánica, es lubricante y tiene baja tensión superficial. Por ello, si se mantiene dentro del conducto radicular durante la conformación, disminuye en gran porcentaje la carga bacteriana y favorece la reparación.

Además, Baasch *et al*. ^24^ añaden que existen dos factores o elementos clave de la irrigación que la hacen mucho más efectiva y, en consecuencia, denotan el éxito del tratamiento y la reparación apical; estos son las agujas a utilizar y la activación del irrigante. Con relación a la aguja, la colocación de su punta más cerca del ápice permite un recambio de la solución más eficiente, lo que tiene como resultado una mejor limpieza y desinfección del conducto radicular. Por otro lado, la distribución del flujo de la irrigación del conducto radicular puede verse afectada adversamente por agujas de diámetros amplios, por grandes distancias entre la punta de la aguja y el tope apical, y por conductos radiculares estrechos. Agregan que, el patrón de flujo de las agujas de punta cerrada es diferente en comparación con las agujas de punta abierta, el flujo es más dirigido a la pared del conducto radicular en lugar del ápice. Por último, de las agujas con extremo abierto, la aguja plana y biselada presentan flujos similares de irrigante a alta velocidad en el conducto radicular ^24^. 

Finalmente, Boutsioukis *et al*. ^25^ analizan la activación del irrigante, o la “irrigación ultrasónica pasiva”, que consiste en realizar movimientos oscilatorios del irrigante dentro del conducto con puntas de ultrasonido, esto con la finalidad de facilitar la llegada de la sustancia a todas las zonas del conducto, mejorar la limpieza y el barrido dentinario, y disminuir la producción del “*vapor look*”, que no es más que la formación de una burbuja de aire dentro del espacio cerrado del conducto radicular.

### La obturación y el sellado tridimensional

En este punto, Vo *et al*. ^26^ destacan que, tanto con la obturación como con la rehabilitación coronal, se busca el sellado completo del sistema de conductos radiculares, con los materiales de obturación de conducto se sella tridimensionalmente, y con la rehabilitación coronal, se sella la entrada de bacterias de la cavidad bucal a los tejidos periapicales. 

Para ello, agregan Bassam *et al*. ^27^, que existe actualmente un sinfín de materiales de obturación y técnicas que garantizan ese sellado, desde cementos resinosos hasta biocerámicos, técnicas de obturación de compactación lateral y vertical, y sistemas de obturación de gutapercha caliente, esto con la finalidad de evitar la llegada de bacterias a los tejidos periapicales y, además, de prevenir reinfecciones o *flear-ups*, garantizan la reparación apical.

### Biología de la reparación perirradicular

Weber *et al*. ^28^ y Siqueira *et al*. ^29^ concuerdan que una vez producido el daño sobre los tejidos periapicales la respuesta inflamatoria inicial es dada por los linfocitos y macrófagos, que responden a bacterias, factores de virulencia y enzimas producidas por las bacterias, ello junto a la activación del sistema de complemento, interleuquinas y la histamina, considerada una sustancia proinflamatoria, favorecen la fagocitosis de los agentes patógenos. 

Así, añaden Weber *et al*. ^30^ y Lampiasi *et al*. ^31^ que esta respuesta inflamatoria que conlleva vasodilatación, aumento de permeabilidad, aumento del líquido en el espacio extracelular y aumento de la presión tisular que aunado a los fenómenos mencionados, ocasiona necrosis del tejido y produce exudado purulento aumentando la respuesta inflamatoria del huésped. Adicionalmente, se produce la activación de los osteoclastos que provienen de la misma línea celular de los macrófagos y esto trae como consecuencia la osteólisis o degradación del hueso, ya que se pierde el equilibrio de la actividad osteoblástica y osteoclástica que mantiene el tejido óseo, de allí la aparición de lesiones osteolíticas perirradiculares.

Ahora bien, como dice Alogaly *et al*. ^32^, cuando el tratamiento endodóntico se realiza bajo condiciones ideales que garantizan su éxito, esto es una buena preparación biomecánica, buen uso de las sustancias irrigantes y una buena obturación con sellado coronal, se está eliminando el factor infeccioso etiológico y creando las condiciones biológicas ideales para la reparación de la región periapical.

Esta reparación, como mencionan Lopes *et al*. ^33^ y Yang *et al*. ^34^ en sus estudios, biológicamente inicia cuando se ha eliminado el factor etiológico y ha culminado la fase inflamatoria; esto permite al organismo dar paso a una fase de remodelación o reparación ósea en la región perirradicular, donde, por acción de fibroblastos y células proinflamatorias, hay formación de tejido de granulación rico en fibras colágenas que dan sostén y facilitan la angiogénesis. Así, desde la periferia se facilita la acción de los osteoblastos que empiezan a producir matriz ósea y forma un tejido osteoide, que posteriormente se va osificando y da paso al remodelado y la formación ósea definitiva.

**Figura 2:**
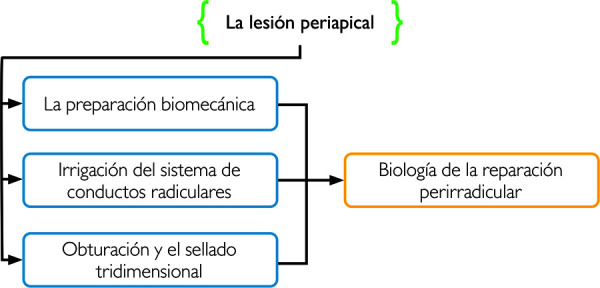
Esquema resumen de aspectos del tratamiento endodóntico no quirúrgico que influyen en la reparación de tejidos perirradiculares

En suma, se visualizaron los procesos reparativos que son consecuencia de un tratamiento endodóntico bien realizado en todos sus procedimientos, lo que incluye un buen diagnóstico, una buena preparación biomecánica y desinfección con sustancias irrigantes, y una buena obturación y restauración coronal, ya que estos son los aspectos claves relacionados al éxito del tratamiento endodóntico no quirúrgico que se traducirá en la reparación de los tejidos perirradiculares. Este análisis, puede sintetizarse en la Figura 2.

## DISCUSIÓN

El factor principal que indica que un tratamiento endodóntico fue exitoso es la reparación de las lesiones periapicales que pueden existir según la patología que el diente presente. Esto se da porque el clínico presta atención a las fases relevantes que implican el tratamiento endodóntico, esto es un buen diagnóstico, una buena preparación biomecánica, una correcta irrigación y uso de las sustancias irrigadoras durante el tratamiento, y un buen sellado del sistema de conductos radiculares en la obturación.

De esta forma, Peters *et al*. ^35^ añaden que se ha documentado una alta prevalencia de dientes con lesiones periapicales y que su diagnóstico debe ser minucioso, junto al diagnóstico pulpar, su correcta evaluación clínica según los signos y síntomas puede establecer un buen pronóstico, aún en el peor de los casos, ya que estas lesiones pueden incluir un gran número de tipos histopatológicos, como son quistes, granulomas y hasta tumores, los cuales si son de etiología infecciosa pueden tener una regresión o curación una vez culminado el tratamiento endodóntico.

Adicionalmente, Tan *et al*. ^36^ dicen que las alteraciones ocurren principalmente en el tercio apical y afectan directamente la región periapical del periodonto, por lo que la necrosis de la membrana periodontal en esta región es bastante probable en casos de necrosis pulpar, es decir, existe una alta carga bacteriana y de toxinas en estas regiones, especialmente en patologías como la periodontitis apical asintomática, que son de larga data y donde es necesaria una desinfección profusa con las sustancias irrigantes para garantizar el éxito del tratamiento endodóntico y la reparación de este tipo de lesiones.

Bien se sabe que son de gran influencia la carga bacteriana y la microbiología en el éxito del tratamiento endodóntico. Esta carga bacteriana se encuentra principalmente en el biofilm intraconducto y es lo que se busca disminuir en mayor medida para garantizar el éxito y posterior reparación de lesiones periapicales. La carga bacteriana es bastante diversa y ha sido descrita por un sinfín de autores ^2^. Puede disminuirse no solo con una efectiva preparación de instrumentos manuales o rotatorios, sino también con el uso efectivo de los irrigantes.

Por otro lado, respecto de la preparación biomecánica, Lima *et al*. ^37^ determinaron la eficacia de la técnica de instrumentación *step-back* en tratamientos endodónticos realizados en una sesión. Ellos trataron 30 dientes con diagnóstico clínico de pulpitis irreversible sintomática y asintomática, y necrosis pulpar, con una variable muy significativa para el pronóstico de reparación de lesiones periapicales como es el dolor posoperatorio.

Para ello, se evaluó el dolor según cuatro criterios numéricos subjetivos para el paciente: ninguno: 0; ligero: entre 1 y 3; moderado: entre 4 y 6; y severo: entre 7 y 10. Además, se consideró que había presencia de inflamación posoperatoria cuando se encontraron dos o más signos clínicos en el diente tratado y el paciente refirió sensación de inflamación o agrandamiento en la zona, sensación de diente crecido u algún síntoma subjetivo referido al proceso inflamatorio.

Se llevaron a cabo controles clínicos y radiográficos a las 72 horas, 7 días y 6 meses, y se observó que el 90 % de los pacientes no padeció dolor posoperatorio, es decir, el 96,67% de los tratamientos fueron eficaces al no existir signos de inflamación o dolor. Por lo que concluyen que la técnica de instrumentación step-back en el tratamiento endodóntico en una sesión fue eficaz en la mayoría de los casos. Por consiguiente, independientemente de una preparación con técnicas manuales o instrumental rotatorio, si esta viene acompañada de una buena irrigación y buen sellado posoperatorio en la obturación, con ausencia de sintomatología después del tratamiento se puede garantizar una reparación de los tejidos periapicales de una forma favorable para el paciente.

Así, aunado a la preparación mecánica, la irrigación juega un rol clave en la desinfección del sistema de conductos radiculares, la cual, bien ejecutada favorece la reparación de los tejidos periapicales ^2^. En este aparte, Guo *et al*. ^38^ compararon la eficacia en la eliminación de la capa de barrillo dentinario o *smear layer* de cuatro técnicas de irrigación diferentes combinadas con NaOCl al 3% a 60 °C y EDTA al 17%.

En el estudio, se tomaron cincuenta dientes unirradiculares que se dividieron aleatoriamente en cinco grupos (n = 10), según los protocolos de activación del irrigante utilizados durante la preparación quimiomecánica: un grupo se irrigó con agujas de salida lateral y activó de forma manual, otro grupo con irrigación ultrasónica, otro grupo con el sistema de irrigación NaviTip FX y otro grupo con EndoActivator; por último, se seleccionó un grupo de control (sin activación). Luego de cada instrumentación, los conductos radiculares se irrigaron con 1 ml de NaOCl al 3% a 60 °C durante 1 minuto, y después de toda la instrumentación, los conductos radiculares se enjuagaron con 1 ml de EDTA al 17% durante 1 minuto. Al mismo tiempo, tanto el NaOCl como el EDTA se activaron con uno de los cinco protocolos de irrigación mencionados y, por último, la eficacia de la eliminación de la capa de barro se calificó en los tercios apical, medio y coronal.

De esta manera, los investigadores mencionan en sus resultados que no hubo diferencias significativas entre el grupo donde se utilizó el sistema de irrigación NaviTip FX, el grupo con EndoActivator y los grupos de control, y cada uno de estos grupos mostró una puntuación más baja que la del grupo con UI. Así, dentro de cada grupo, los tres tercios se clasificaron en el siguiente orden: coronal > medio > apical. En el tercio coronal, el grupo NaviTip FX fue mejor la remoción del *smear layer* que el grupo donde se utilizó UI. En el tercio medio y apical las diferencias no fueron significativas entre ninguno de los grupos.

En consecuencia, se concluyó que la combinación de NaOCl al 3% a 60 °C y EDTA al 17% pudo eliminar la capa de barrillo de manera efectiva, similar al sistema NaviTip FX o al EndoActivator, y estos tres protocolos fueron más efectivos que la UI.

Así, se entiende que una buena remoción de la capa de barrillo dentinario, independiente de la técnica utilizada la para la irrigación y su activación, garantiza el éxito del tratamiento endodóntico y la reparación de los tejidos perirradiculares.

En adición, Kaushal *et al*. ^39^ determinaron cuál irrigante remueve efectivamente el smear layer de los tercios coronales, medios y apicales del conducto radicular. Ellos estudiaron 120 dientes monorradiculares premolares mandibulares, que dividieron en 4 grupos de 30 dientes cada uno: un grupo irrigado con 5 ml de EDTA al 17%, otro grupo irrigado con ácido cítrico al 10%, otro grupo irrigado con ácido maleico al 7% y otro grupo irrigado con solución salina (grupo control), todos preparados con instrumentación manual hasta lima tipo K #40,02.

De este estudio se extrae que el ácido maleico al 7% y el ácido cítrico al 10% son igualmente eficaces en la eliminación del barrillo dentinario del tercio coronal y medio, pero en el tercio apical, el ácido maleico al 7% es más eficaz que el ácido cítrico al 10%. Entre el ácido cítrico y el EDTA, ambos son igualmente efectivos en la eliminación del barrillo dentinario del tercio coronal y medio, pero, en el tercio apical, el ácido cítrico al 10% es más eficaz que el EDTA al 17%.

Estudios como este denotan la implicación no solo de la técnica utilizada en la irrigación, sino de la sustancia irrigante, la cual, siempre y cuando garantice una buena eliminación del barrillo dentinario y desinfecte el conducto disminuyendo su carga bacteriana, podrá brindar una buena reparación de los tejidos periapicales y un tratamiento endodóntico exitoso y asintomático después de la intervención.

Entonces, Tabassum *et al*. ^40^ añaden que el fracaso endodóntico se produce principalmente por la persistencia de bacterias (intra y extraconducto), el llenado inadecuado del conducto (limpios, desinfectados y obturados), las sobreextensiones de materiales de obturación radicular, el sellado coronal inadecuado, los conductos no tratados (tanto principales como accesorios), los errores de procedimiento iatrogénicos y las complicaciones de la instrumentación (escalones, perforaciones o instrumentos separados).

En suma, el objetivo del tratamiento endodóntico es el desbridamiento y la limpieza minuciosa del sistema de conductos radiculares de cualquier tejido pulpar infectado, para que se pueda moldear el espacio del conducto y prepararlo para rellenarlo con un material inerte, lo que previene o minimiza cualquier posibilidad de reinfección, y favorece la reparación de los tejidos perirradiculares ^40^. 

Holland *et al*. ^41^ mencionan que la reparación completa solo se produce cuando el antígeno o agente causal ha sido neutralizado durante la respuesta inflamatoria, por ejemplo, la periodontitis apical sintomática. Así, en la infección pulpar, el cierre del suministro sanguíneo al conducto radicular beneficia la proliferación bacteriana. Además, favorece la inflamación periapical para neutralizar el antígeno. Esta respuesta inflamatoria ocasiona, además, resorción ósea donde se crea el espacio para la infiltración de células inmunes, que luego se organizan en una barrera para secuestrar la infección.

Esta resorción ósea y la consecuente formación ósea -si se crean las condiciones adecuadas- son procesos que implican la actividad de osteoclastos, osteoblastos y osteocitos, que se ven afectados por condiciones sistémicas y locales ^42^. Sin embargo, la homeostasis ósea se altera durante la periodontitis apical, lo que favorece las resorciones óseas. Por ello, la aplicación de todos los protocolos clínicos existentes debe considerar las condiciones locales y sistémicas que contribuyen al proceso de curación.

### Limitaciones

Aunque existen numerosos estudios que desarrollan y analizan la reparación de los tejidos perirradiculares en el tratamiento endodóntico, la mayoría de estos tienen un enfoque biológico y explican el proceso de reparación con un enfoque histológico y celular, es decir, explican los procesos celulares que se producen en la reparación y regeneración ósea, una vez culminado el tratamiento.

De esta forma, existió la limitante de conseguir estudios que explicaran el efecto biológico y la relación de cada uno de los pasos que se dan en un tratamiento endodóntico para conseguir esa reparación. Esos pasos comprenden la preparación biomecánica, la irrigación y desinfección del sistema de conductos radiculares y la obturación y sellado coronal del sistema de conductos radiculares.

Así, a partir de esta investigación se pueden realizar estudios que analicen la relación de estos aspectos con la reparación periapical.

## CONCLUSIÓN

El éxito del tratamiento endodóntico radica principalmente en la ausencia de sintomatología y de signos radiográficos de infección, esto se consigue con una buena aplicación de protocolos y procedimientos clínicos enfocados en la desinfección del sistema de conductos radiculares, desde el momento del diagnóstico hasta la obturación, sin dejar de lado la preparación biomecánica e irrigación del sistema de conductos radiculares, así como la rehabilitación del diente desde el punto de visto estético o protésico.

La reparación de los tejidos periapicales ocurre meses y años después del tratamiento, una vez que, mediante el mismo, se hayan brindado las condiciones para la regeneración ósea; en esto consiste una buena desinfección del sistema de conductos radiculares.
